# Functional near-infrared spectroscopy for monitoring macaque cerebral motor activity during voluntary movements without head fixation

**DOI:** 10.1038/s41598-018-30416-7

**Published:** 2018-08-09

**Authors:** Toru Yamada, Hiroshi Kawaguchi, Junpei Kato, Keiji Matsuda, Noriyuki Higo

**Affiliations:** 1 Human Informatics Research Institute, National Institute of Advanced Industrial Science and Technology (AIST), Tsukuba, Japan; 2 Graduate School of Comprehensive Human Sciences, University of Tsukuba, Tsukuba, Japan

## Abstract

We developed an fNIRS system for monitoring macaque cerebral motor activity during voluntary movements without head fixation. fNIRS data at 27 channels in 7.5 mm spatial interval were calibrated by simulating light propagation through the macaque cranial tissues. The subject was instructed to repeatedly (75 times) retrieve a food pellet with alternating left or right hands from a food well for each session. We detected significant increases in oxygenated hemoglobin (Hb) and decrease in deoxygenated Hb in the primary motor area (M1) contralateral to the hand used. In more rostral and ventral regions in both hemispheres, the hemodynamic similarly changed regardless of used hand. Direct feeding to the mouth eliminated activity in the hand M1 whereas that at bilateral ventral regions (mouth M1 area) remained. Statistical analyses for the hemodynamics between left/right-hand use revealed the location of each hand M1 in either hemisphere. In these regions, the maximum amplitude and time of the maximum amplitude in the hemodynamic response evoked by food retrieval were highly correlated with the time associated with food retrieval. We could assign each channel to an appropriate functional motor area, providing proof of principle for future studies involving brain damage models in freely moving macaque monkeys.

## Introduction

Brain damage, such as that induced by stroke, often causes functional deficits that can recover to varying degrees thereafter. During the recovery process, neurorehabilitation training promotes neuroplastic changes that can lead to functional recovery^1–3^. However, although numerous clinical trials are currently focused on improving neurological outcomes, the effect of rehabilitative training is largely unclear. In these trials, it is particularly difficult to control for differences in the position, extent, and the elapsed time from injury. Animal model studies in which damage is induced in specific regions of the brain, under controlled conditions, circumvent this issue, enabling more quantitative analyses. In particular, studies of nonhuman primates have the advantage of potentially translating to human patients, because their brains, body structures, and genetic backgrounds are closely similar across primate species^4–6^. One particularly relevant issue following brain damage relates to hand functionality, which is essential for quality of life as hand dysfunction seriously impairs the ability to perform day-to-day tasks. In some nonhuman primate species, including macaque monkeys, dexterous hand function is as highly developed as in humans^7–11^, making them an ideal model for the investigation of hand movement following brain damage.

We previously reported that macaque monkeys with induced primary motor area (M1) lesions exhibited recovery of dexterous hand movements after intensive rehabilitative training^12^. The ability to monitor brain activity is essential to understanding the mechanisms that underlie rehabilitative training-induced functional recovery. We imaged the brains of awake monkeys using H_2_^15^O positron emission tomography (PET) scans during hand movements and showed that activity in the ventral premotor cortex increased during the recovery period, suggesting that this cortical area contributes to the recovery of motor function after an M1 lesion^13^. However, PET requires the head to be fixed during scanning, inhibiting natural movement and potentially affecting the results. Moreover, the need to use a radioactive tracer impedes frequent monitoring using PET.

Functional near-infrared spectroscopy (fNIRS) measures the hemodynamic response evoked by functional cerebral activation. Because light travel is limited through tissue, optimization of the location of the source–detector optode pairs allows for high spatial selectivity of fNIRS^14^. A multichannel array with an appropriate channel interval (15 mm) enables sufficient spatial resolution to discriminate human M1 from other motor-related areas^15,16^. To adapt this design for a macaque brain requires proper accounting of the smaller size of its head and brain.

Signal contamination of fNIRS measurements is a key concern for accurate quantitative monitoring of the hemodynamic response. In general, fNIRS signal contaminants include experimental errors and systemic physiological fluctuations that can occur in excess of instrumental noise^17^. Experimental error is typically caused by optode movement due to insufficient fixation. Optodes are generally held to the subject’s head using some fixed holder, and if this holder is unstable, a subject’s movements can alter the optode-scalp gap distance causing artifacts in fNIRS signals. These artifacts exhibit slower baseline fluctuation including drift and level shift after spiky peak^18,19^. During the study of awake monkeys, subjects may move their heads and/or change postures in association with task execution. Also, as the scalp is intrinsically stretchable and can shift, optode fixation is invariably unstable. In total, movement-induced optode artifacts can cause severe distortions of the shape of hemodynamic responses. Furthermore, blood flow changes in the scalp tissue are a major source of systemic physiological fluctuation-induced fNIRS artifacts^20–22^. This is because blood flow fluctuates with changes in respiration, blood pressure, posture, and vasomotor reactions in association with certain psychophysiological responses.

In the present study, we developed a new fNIRS system for monitoring macaque cerebral motor activity during voluntary movements without head fixation. We applied our previously developed method for calculating the light propagation in optical models^14^ to optimize the optodes’ distance and arrangement in the macaque motor-related cortical region. We used the most direct way to address both the need for absolute spatial fixation of optode placement as well as the elimination of systemic physiological fluctuations by directly affixing optodes to the skull surface. On the basis of the spatial and temporal differences in the hemodynamic responses obtained, we were able to assign each measurement channel to an appropriate functional motor area, providing a proof of principle for future studies involving brain damage models in freely moving macaque monkeys. The study was comprised of three parts. First, anatomical data of three macaque monkeys were subjected for optimization of the fNIRS measurement condition. Second, for a subject in the three monkeys, fNIRS optodes were arranged on the skull surface and the fNIRS signal sensitivities was calculated. And third, the fNIRS measurement during behavior experiments was conducted and the data were analyzed. Overview of this study was shown in Supplementary Fig. S1.

## Methods

### Animal Care and Use Committee approval

The protocol of the present study was approved by the Institutional Animal Care and Use Committee of the National Institute of Advanced Industrial Science and Technology, Japan, and was carried out in accordance with the guidelines within the “Guide for the Care and Use of Laboratory animals” (Eighth ed., National Academy of Sciences).

### Optimization of fNIRS optode distance in monkey’s motor activity measurement

The fNIRS optode arrangement was customized for the present monkey study. Because the superficial layers such as the scalp and skull of the monkey head are thinner and the brain is smaller than those of humans, the optimal source–detector distance for measuring fNIRS signals from the monkey brain was presupposed to be smaller. We estimated the source–detector distance optimal for monkey brains through a calculation simulating light propagation in an optical model of the monkey head.

For this simulation, T1 and T2 weighted magnetic resonance (MR) head images of three macaque monkeys including the trained subject were used and optical models of these heads were constructed. Based on simulations over the three individual models, universality of the calculation results were examined. Each model consisted of six optical layers: air, skull, cerebrospinal fluid, gray matter, white matter, and other soft tissues. T1 and T2 weighted MR images present sufficient contrasts among these layers to identify each^23^. In the MR image of the trained (scalp-incised) subject after forming optode sockets, the skull surface was not clear because the skull tissue and the socket material (acryl resin) contain few protons and thus have little T1 resonance. In this case, the skull surface was presumed to be identical to the boundary between the scalp and skull in the image before forming the sockets (i.e., the image of the intact subject’s head). The position of this boundary in the post-implantation image was identified by matching the before and after brain tissue boundaries with each other. Overlapping was executed through a rigid body transformation (FSL 5.0, Oxford, UK). The boundaries of other tissues and the optode positions were directly identified using the image after forming the sockets. In cases of other two monkeys, six-layered optical models including soft tissue layer were firstly constructed based on MR images of intact heads. Thereafter, for uniforming layered structures in these models with the scalp-incised model, the superficial soft tissue layer of parietal region was replaced by air layer. The coefficients of absorption and reduced scattering in each tissue at 800 nm wavelength were taken from literature^24–27^. A refractive index of *n* = 1.40 was used for all tissue layers. Light propagation in the models was calculated using the diffusion equation. The finite element method was conducted to solve the diffusion equation^28^. In the MR images of each monkey, the center position of the hand knob, known as the hand motor center in the M1 area was identified manually by a researcher well-versed in monkey brain anatomy. The position of the hand knob in the optical model was projected onto the scalp surface. Source–detector pairs separated by 5 to 25 mm (in 5 mm steps) were introduced to the model so that the midpoint of each source–detector pair aligned with the projected point (i.e., the hand knob). Optode pairs aligned both parallel and orthogonal to the central sulcus adjacent to the hand knob were examined. In each condition, the detected light intensity, the mean optical path length^29^, and spatial sensitivity profile (SSP)^30^ were calculated. The SSP over all the voxels in the model was obtained through the calculation of the photon measurement density function in each voxel and was normalized by mean optical path length. The photon measurement density function provides the probability of light transit through the voxel in a given source–detector condition and was calculated on the basis of the reciprocity principle^14,31^. Because the detected light travels through each voxel with different sensitivity, the sum of the SSP of voxels within a certain layer is equivalent to the partial optical path length in that layer. For example, in this calculation, the partial optical path length in the gray matter (L_gray_) was calculated as the sum of SSP in the corresponding gray matter. To evaluate the effective signal sensitivity for cerebral motor activity, a regional L_gray_ in a cubic volume of 5.4 × 5.4 × 5.4 mm^3^ at the hand knob was also calculated. Further details in the simulation calculation are described elsewhere^32^.

**Figure 1:**
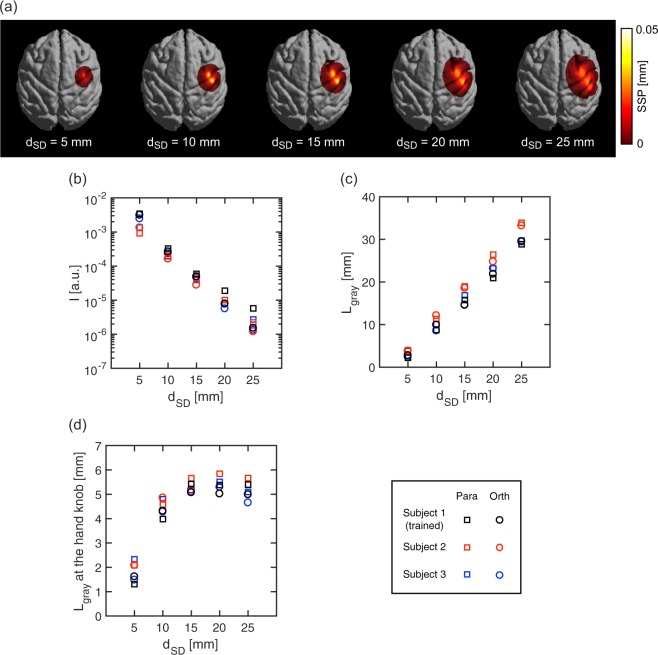
Simulation calculations of light propagation through head tissues of monkey head models (**a**) Spatial sensitivity profile (SSP) of a macaque monkey (Subject 1) in orthogonal optode alignment for source–detector distances, d_SD_, of 5, 10, 15, 20, and 25 mm. (**b**) Detected intensity, I, versus d_SD_. (**c**) Partial optical path length in the gray matter layer, L_gray_ versus d_SD_. (**d**) Regional L_gray_ at the hand knob versus d_SD_. Optode pairs aligned both parallel (Para) and orthogonal (Orth) to the central sulcus adjacent to the hand knob were examined. Subject 1 is the trained monkey to be measured with fNIRS.

Results of the calculation are shown in Fig. 1. The SSP on the gray matter surface became broader as the source–detector distance (d_SD_) increased (Fig. 1a), which indicated that shorter distance provided higher spatial resolution but lower detection sensitivity. Similar dependencies of SSP on d_SD_ were observed in all subjects and parallel and orthogonal optode alignment conditions. The predicted detected intensity (I) monotonically decreased with increase in d_SD_ (Fig. 1b) and L_gray_ monotonically increased (Fig. 1c). However, the regional L_gray_ at hand knob asymptotically approached to a finite length (Fig. 1d). The predicted values in each calculation were very similar among the cases of subjects and optode alignment conditions. In Fig. 1d, the regional L_gray_ showed little increase in the range of d_SD_ longer than 15 mm. This indicates that the signal sensitivity for the gray matter in the hand knob region is not gained by increasing d_SD_ more than 15 mm. On the other hand, the L_gray_ in all over the inter-optode region further increased in this range (Fig. 1c). This indicates the increase in signal contribution from regions other than hand knob, namely, the degradation in spatial resolution of the signal detection in the range more than 20 mm. By taking consideration of these results, we concluded that 15 mm is most appropriate for d_SD_ to detect activity changes in the macaque motor cortex, in which motor representations of the face, hand and arm are located with difference of several millimeters in M1^33^.

### Surgical procedures

One healthy adult Japanese macaque monkey (female; 5.0 kg) without any history of experimentation was used. The positions of M1 and the premotor area (PMA) were determined using stereotaxic coordinates from MR images of the monkey’s brain using a 3.0 T MR imaging (MRI) system (Philips Ingenia 3.0 T, Philips Healthcare, Best, The Netherlands). The anatomical MRI protocols consisted of a T1-weighted turbo field echo sequence (repetition time/echo time, 7.3/3.2 ms; number of excitations, 2; flip angle, 8°; field of view, 134 × 134 mm; matrix, 224 × 224; slice thickness, 0.6 mm; number of slices, 200). Pentobarbital anesthesia was administered at 20 mg/kg, after which the parietal region of scalp was incised and optode sockets were formed on the skull surface with self-curing acrylic resin (UNIFAST II Clear, GC Corporation, Tokyo, Japan). Titanium oxide (KA-30, Titan Kogyo, Ltd., Ube, Japan) was mixed into the resin at a weight ratio of 1:450 to match its optical scattering property to that of the skull. These procedures were done under sterile conditions.

### Optode arrangement at parietal region of the monkey head

**Figure 2:**
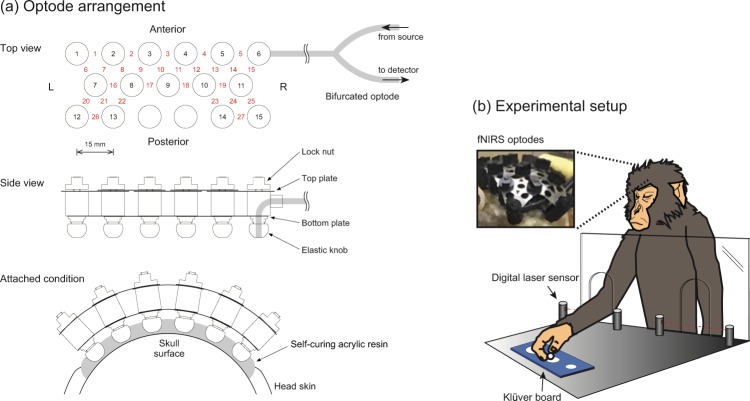
(**a**) Schematic illustration of the triangular bidirectional optode arrangement and the holding system. In the top view, open circles with black-colored numbers indicate optode positions. Red-colored numbers between optodes indicate channel positions. L, and R represent left side and right side of optode arrangements on the subject’s head, respectively. Adjacent optodes were cross-linked by two-layered linkage plates. The lower plates hold the distances between optodes. The upper plates were equipped with elongated holes and nuts. After the optode tips of globular shape were inset into the sockets on the acrylic resin, the optode arrangement was consolidated by locking nuts. (**b**) Experimental setup for the macaque’s food retrieval task. The macaque monkey retrieved spherical food pellets (5 mm in diameter) from cylindrical wells (20, 11, and 10 mm in diameter) on a Klüver board. A digital laser sensor was installed in front of the Klüver board to detect the initiation and duration of the reach-to-grasp movement.

For discriminating the motor representation in M1 with fNIRS signals, the spatial interval between channels in fNIRS should be several millimeters to discriminate the hemodynamic responses at different gyri, whereas the source–detector distance of 15 mm is required for appropriately detecting gray matter responses. To satisfy both conditions, optodes (about 10 mm in diameter) placed conventionally would have to be very densely packed. We therefore adopted a triangular bidirectional optode arrangement, in which the optodes are placed at regular triangle lattice points, and each is used as a source and a detector through temporal switching^16^. The schematic illustration of the arrangement is shown in Fig. 2a (Top view). As determined above, the source–detector distance was fixed to be 15 mm; thus, the spatial interval among adjacent channels was 7.5 mm. Each optode has bifurcated ends connected to a source or a detector in the fNIRS OMM-3000 system (Shimadzu Corporation, Japan). The bifurcated optodes were custom made of optical fiber bundles (Moritex Corporation, Japan). Using ternary optodes as one source and two detectors, signals from two channels are collected simultaneously, and by completing illumination at all optodes, two measurements for every channel are accomplished.

As shown in Fig. 2a (Side view), fifteen optodes held with a custom holder were fixed into the previously implanted socket wells on the skull surface just before every experimental session. There, adjacent optodes were cross-linked by two-layered linkage plates. The lower plates hold the distances between optodes as 15 mm. The upper plates were equipped with elongated holes and nuts. After the optode tips of globular shape were inset into the sockets, the optode arrangement was consolidated with locking nuts. Switching between illumination and detection in each optode was controlled through software built into the OMM-3000. Twenty-seven channels were available for measurement in the parietal region of the monkey head. Optical absorbance data at wavelengths of 780, 805, and 830 nm for each channel and the digital signal from the Digital Laser Sensor were recorded by the OMM-3000.

We then calculated light propagation in the subject’s head at wavelengths of 780, 805, and 830 nm for this optode arrangement. For constructing an optical model including the optodes’ precise positions, T1 weighted MR images of the subject both before and after implantation of the optode sockets were obtained. After, images were obtained by filling the socket wells with an MR marker (Cu-sulfate solution). On the basis of this optical model, SSP and the optical partial path length at each wavelength at each channel were calculated in the same way as described above.

**Figure 3:**
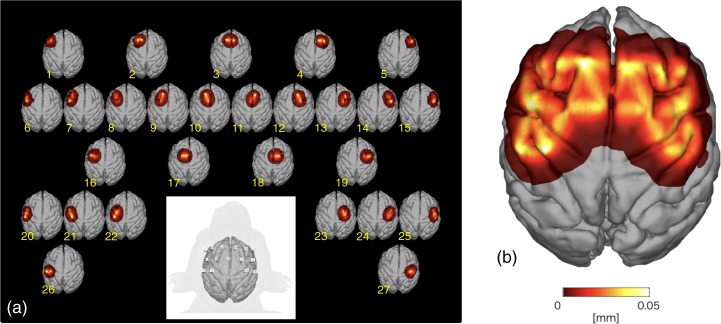
Spatial sensitivity profile (SSP) in the subject monkey. (**a**) SSP of the measurement channels in the subject monkey. (**b**) The maximum value of each simulated voxel over the SSPs of all channels.

Figure 3a shows the image of subject’s brain with optodes (lower center) and SSPs at the wavelength of 805 nm at 27 channels. In general, the SSPs of adjacent channels overlapped indicating that the optode arrangement offers nearly complete coverage of the underlying gray matter layer. The maximum value of SSPs from all of the channels was calculated (Fig. 3b), allowing for demarcation of areas of higher and lower sensitivities. For appropriately comparing hemodynamic responses among the channels, the fNIRS signals were calibrated using the estimated optical partial path length at each wavelength at each channel.

One hundred thirty milliseconds were required to complete illumination at all optodes while recording two measurements for every channel. We used the average of the two measured absorbance data as one sample in a temporal resolution of 130 ms. These absorbance data were calibrated using the optical partial path length estimated through the simulation described above. Baseline drifts in the calibrated absorbance data were removed by a third polynomial fitting. High-frequency noise was filtered using a fourth Butterworth-type filter at 0.7 Hz.

The concentration changes in oxygenated and deoxygenated Hb, ΔHbO and ΔHbR, respectively, were transformed from the calibrated absorbance data at each measurement wavelength on the basis of the modified Beer–Lambert law. The molar absorption coefficients of each Hb species in the translation matrix were taken from a literature^34^.

### Event-related experiment of food retrieval task

The monkey was trained a small-object retrieval task closely resembling that used in our previous studies^12,35^. In this task, the monkey sat in the primate chair and retrieved a small spherical food pellet (5 mm in diameter) from the Klüver board, which contained cylindrical wells of three different sizes (10, 11, and 20 mm in diameter, 5 mm in depth). The Klüver board was located at the monkey’s waist height and at a sagittal distance of about 300 mm from the monkey’s shoulder. The size of the well was fixed within a daily session, and the monkey retrieved food pellets approximately every 20 s using each hand alternately. One hundred fifty trials (75 for each hand) were performed in each daily session. To observe behavioral changes during the learning of food retrieval, two separate series of food retrieval sessions were executed. In both the session series, a food well of 11 mm in diameter was used for the initial session. Food wells of 20 and 10 mm were separately used for three additional sessions in each session series. Each session series was completed within a week. More than several weeks were left between the two session series.

Two additional sessions were also performed: (1) food retrieval only by left hand in 75 trials in which the subject’s right forelimb was constrained, (2) direct feeding to the mouth by experimenter in 75 trials with both forelimbs constrained. Cross-validation of potential contaminants in the fNIRS signals was conducted more than several weeks before of the start of the testing sessions and was performed using left and right hands alternatively.

The onset and end time points of food retrieval movement by either the left or right hand were detected by a Digital Laser Sensor (LV-11SB with sensor head LV-S72, Keyence, Osaka, Japan) installed in slits within the acrylic resin board located between the primate chair and the Klüver board (Fig. 2b). These retrieval event data were obtained concurrently with the fNIRS signals and were used for event-related signal analyses.

### Statistical analysis

As described in the previous section, the onset and end time points of food retrieval movement by the left or right hand were monitored with the Digital Laser Sensor. The signal from the Digital Laser Sensor was received and recorded with the OMM-3000 concurrently with the fNIRS absorbance data. On the basis of this retrieval event data, the times of onset and duration of each retrieval event in each experimental trial were measured and used for the event-related signal analyses described below.

The durations of food retrieval events in 75 trials of each left- and right-hand use in each experimental session were sampled. For normalizing the sample distributions, the Box–Cox transformation with λ = −1 was applied for each session data. The homoscedasticity among the transformed distributions was examined with Bartlett’s test. Differences among the sample averages of different sessions and used hands were compared using analysis of variance (ANOVA) with correction for multiple comparisons using the Tukey–Kramer method.

**Figure 4:**
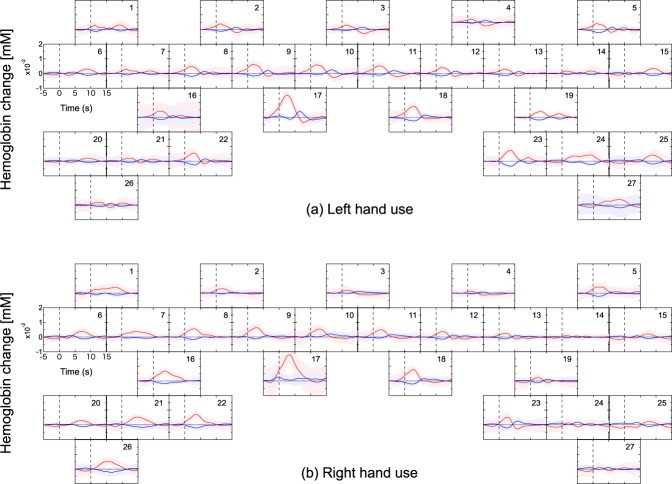
Representative hemodynamic response during a food retrieval experimental session. (**a**) Left-hand use and (**b**) right-hand use. Each frame depicts the block averages and the standard deviations of each channel among for ΔHbO and ΔHbR drawn with red and blue lines and bands, respectively.

**Figure 5:**
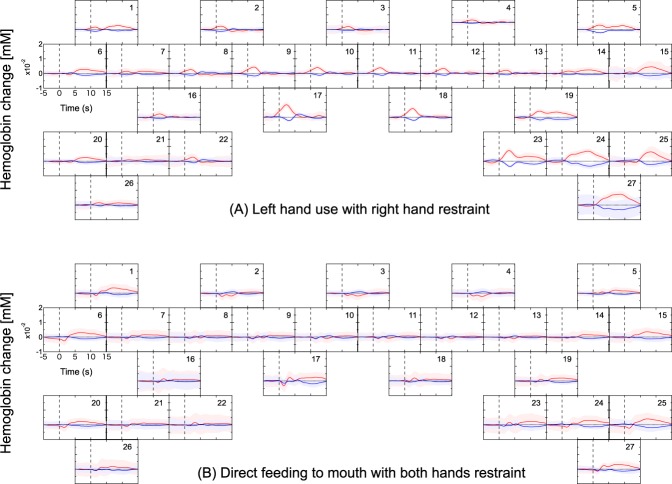
Hemodynamic responses evoked by different restrained conditions. (**A**) Left hand food retrieval with the right forelimb constrained and (**B**) direct feeding to mouth with both hands restrained. Each frame depicts the block averages and the standard deviations of each channel among for ΔHbO and ΔHbR drawn with red and blue lines and bands, respectively.

**Figure 6:**
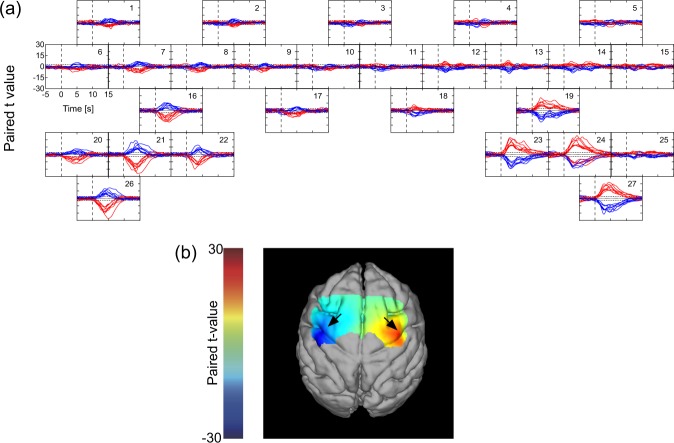
(**a**) Time course curves of paired *t*-values of hemodynamic response. The *t*-values for the paired *t*-test were calculated from the difference in hemodynamics during trials of left-hand and right-hand use. The *t*-values of ΔHbO and ΔHbR for each of the eight sessions were drawn with red and blue lines, respectively. The dotted lines in each frame indicate levels of *p* = 0.05. (**b**) Color-mapping of *t*-values onto the subject’s anatomical MRI. The *t*-values for ΔHbO of the first session using a food well of 20 mm 5 s after task onset are depicted. Arrows indicate the positions of “hand knob” in each hemisphere.

The fNIRS data in each of the 150 trials (75 right hand, 75 left hand) was time-locked (t = 0) to the onset of food retrieval movement for that trial. Then the values of ΔHbO and ΔHbR for each time point (*i.e*., a 130 ms period, starting from t = 0 for each trial) were averaged and plotted in Fig. 4 (and in Fig. 5 for additional tasks). For each time point, a paired *t*-test between the left-hand trials and right-hand trials was also conducted. Figure 6 plots the *t*-score values for the contrast of left versus right hand movement, for both ΔHbO and ΔHbR, at each time point in each of the eight sessions. When significant amplitude (*p* < 0.05) in the *t*-value curves was detected, the maximum amplitude in the ΔHbO and ΔHbR curves and the time elapsed from onset to maximum amplitude was calculated. These calculations at each channel were performed for each experimental session. Maximum amplitude and elapsed time in each session were averaged between channels for each brain hemisphere separately and the Pearson’s product-moment correlation coefficient of them against the time required for food retrieval were calculated.

## Results

### Hemodynamic response evoked by task execution

The representative block averages of ΔHbO and ΔHbR evoked by food retrieval with left-hand and right-hand uses are shown in Fig. 4. In general, the ΔHbO and ΔHbR exhibited opposite directional changes (i.e., an increase in ΔHbO was accompanied with a decrease in ΔHbR, and vice versa). Larger ΔHbO and ΔHbR were recorded from channels in hemispheres contralateral to the hand being used. For example, channels 16, 21, 22, and 26 placed in the left hemisphere recorded the largest changes when the right hand was used. Similarly, channels 19, 23, 24, and 27 located in the right hemisphere indicated markedly larger amplitudes when the left hand was used. In the other channels, similar left/right ΔHbO and ΔHbR amplitudes were observed regardless of which hand was being used.

To further clarify the channels that indicated changes in ΔHbO and ΔHbR that were either dependent or independent of the hand used, additional retrieval tasks were performed in which the right forelimb was restrained during left-handed food retrieval (Fig. 5A). Interestingly, left hand use with right hand restraint caused changes in ΔHbO and ΔHbR to entirely disappear from left hemisphere channels that had previously indicated used-hand-dependent signals, whereas those at the channels of symmetrical positions in right hemisphere remained unchanged versus unrestrained. Channels that indicated used-hand-independent signals, however, exhibited similar ΔHbO and ΔHbR levels to those observed in the corresponding channels during the unrestrained sessions.

To closely examine used-hand-independent signals, a further task was conducted where both forelimbs were constrained and food pellets were fed directly to the subject’s mouth by an experimenter. Feeding via the mouth blunted the appearance of the changes observed in the above identified used-hand-dependent regions as well as rostral regions of both sides (Fig. 5B vs. Figs 4 and 5A). Interestingly, however, the most ventral regions of both hemispheres exhibited changes in ΔHbO and ΔHbR that were similar to those in which a hand was used (Figs 4 and 5A).

### Characteristic hemodynamic responses in the motor-related cortex

To statistically evaluate the difference in Hb changes between left- and right-hand uses, paired *t*-tests were conducted for each Hb species for each channel in each session. Time course curves of *t*-values for ΔHbO and ΔHbR at each channel are shown in Fig. 6a. Channels that recorded statistically significant amplitudes of *t*-value in ΔHbO and ΔHbR (*p* < 0.05) were highly localized to the caudal region of both hemispheres. During right-handed movement, channels in the left hemisphere (Ch 16, 21, 22, and 26) recorded *t*-values for ΔHbO and ΔHbR that were negative and positive, respectively. Correspondingly, those channels in the right hemisphere (Ch 19, 23, 24, and 27) exhibited opposite directional features. The shape and amplitude of *t*-values for each Hb species were very nearly inversely correlated with respect to their localizations in the left and right hemispheres. In Fig. 6b, the *t*-values for ΔHbO from a first session using a 20 mm food well from each channel 5 s after task onset were color-mapped and superimposed upon the anatomical MR image. Foci indicate negative and positive *t*-values of the hand knobs of the left and right M1, respectively. These representative results were similar to those obtained during other sessions. From these findings, the region at Ch 16, 21, 22, and 26, and the region at Ch 19, 23, 24, and 27 were identified as the hand M1 in the left and right cerebral cortices, respectively.

The hemodynamic responses associated with task execution shown in Figs 4 and 5 generally adhered to specific reproducible patterns. In several instances, hemodynamic responses were detected before the onset of hand movement. This may be due to a time lag of the detection of forelimb by the laser sensor. In other instances, responses were observed after some latency from task onset. These responses were observed in a more ventral region than the hand M1 area (Figs 4 and 5), occurred bilaterally regardless of the side of hand use, and of note, were the only to remain when the subject’s hands were both immobilized and the subject was fed directly (Fig. 5B). One likely explanation is that food retrieval/feeding was always followed by eating, which is associated with bilateral mouth movements. Indeed, a paired *t*-test indicated that there were no inherent bilateral statistical differences between these regions (Fig. 6a). On the basis of these findings, the bilateral ventral regions at channels 1, 5, 6, 15, 20, and 25 were identified as the mouth M1 area, which is located ventrally to the hand M1 area^33^.

**Figure 7:**
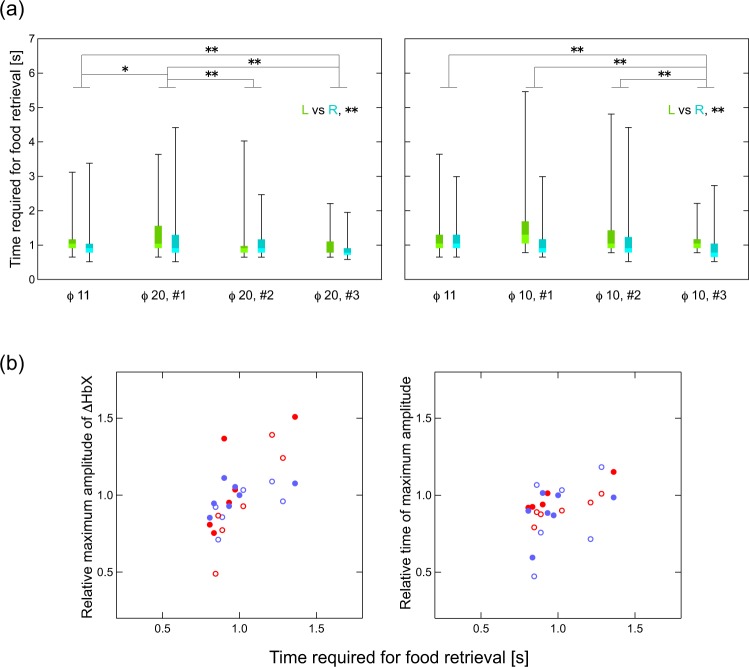
(**a**) Boxplots of the time required for food retrieval of 75 trials at each session. Left and right frames represent session series using food well sizes of 20 and 10 mm, respectively, each starting with a well size of 11 mm. In each session, plots of trials with left and right hands were colored with green and blue, respectively. The marks * and ** indicate statistical significance of *p* < 0.05 and *p* < 0.01, respectively. (**b**) Correlation between hemodynamic responses and the time required for food retrieval. Left panel, maximum amplitudes in hemodynamic response versus time required for food retrieval; right panel, time of maximum amplitude in hemodynamic response versus time required for food retrievals. Closed red circles indicate ΔHbO in the left hemisphere; closed blue circles indicate ΔHbR in the left hemisphere; open red circles indicate ΔHbO in the right hemisphere; and open blue circles indicate ΔHbR in the right hemisphere.

The channels in a more rostral region than the M1 indicated hemodynamic responses that began before the task onset. These responses were not observed when food was passively eaten after being directly fed (both hands restrained). Also, the responses were distinctively different from those observed in the hand M1 area, indicating no significant laterality associated with the used hand. It is known that bilateral PMAs are activated during forelimb movement regardless of the side of hand actually used^36^. From these findings, the relatively rostral dorsal region in both hemispheres at channels 8, 9, 10, 11, 12, and 13 were identified as PMA. The channels in the most medial dorsal region (channels 17 and 18) should be assigned as supplementary motor area (SMA).

To quantitatively evaluate different tendencies in block-averaged hemodynamic responses in different regions, the times of maximum amplitude in the hemodynamic responses (representatively shown in Fig. 4) were compared over different regions. The averaged times of maximum amplitude over sessions were 0.71 ± 0.47 s for ΔHbO and 0.55 ± 0.65 s for ΔHbR in PMA, 2.43 ± 0.84 s for ΔHbO and 2.48 ± 1.43 s for ΔHbR in hand M1, and 6.63 ± 0.88 s for ΔHbO and 7.05 ± 0.89 s for ΔHbR in mouth M1. ANOVA with correction for multiple comparisons using the Tukey–Kramer method showed significant differences of *p* < 0.01 between PMA and hand M1, hand M1 and mouth M1, and mouth M1 and PMA. Difference between ΔHbO and ΔHbR was not significant (*p* > 0.1). Taken together, the data indicate that using our system, hemodynamic responses characteristic of each motor-related cortex were obtained with high sensitivity and fidelity. The correspondence between the cerebral area and the measurement channels were shown in Table 1.

**Table 1:** The correspondence between the cerebral area and the measurement channels.

	Left hemisphere	Right hemisphere
PMA	Ch 8, 9, and 10	Ch 11, 12, and 13
SMA	Ch 17	Ch 18
Hand M1	Ch 16, 21, 22, and 26	Ch 19, 23, 24, and 27
Mouth M1	Ch 1, 6, and 20	Ch 5, 15, and 25

### Comparison of hemodynamics with behavioral measurement

The time required for food retrieval in 75 trials from each session for each hand is shown in Fig. 7a. In both session series, using food wells of 20 and 10 mm, the time required for food retrieval increased once when the size of the food well was changed from the initial size of 11 mm; thereafter, time decreased as training progressed. As expected, time of retrieval was significantly different between the right and left hand (*p* < 0.01).

We hypothesized that differences in retrieval time would be associated with differential activation of the corresponding cerebral region. To examine this possibility, we focused on those channels that were previously associated with hand use (Fig. 4), namely, channels 16, 21, 22, and 26 in the left hemisphere and channels 19, 23, 24, and 27 in the right hemisphere. Maximum amplitudes (either peaks or valleys) in the time course curves of ΔHbO and ΔHbR and the times of the maximum amplitudes were calculated for each session and averaged over the channels in each hemisphere. These averages were plotted against time of food retrieval (normalized to the time of retrieval of the initial session) (Fig. 7b). Pearson’s product-moment correlation coefficients of the time for food retrieval to the maximum amplitudes and the time of maximum amplitudes were calculated for each Hb species and each hemisphere (see Supplementary Table S1). Significantly high positive correlations between the maximum amplitude and the time for retrieval were found for ΔHbO in both hemispheres.

### Correlation between ΔHbO and ΔHbR in the cerebral hemodynamic response

The relationship between ΔHbO and ΔHbR in the hemodynamic response to the food retrieval task at each channel over all the sessions were plotted in Supplementary Fig. S2. Regardless of the cases in left (green dots) and right hand use (blue dots), distinctive linear relationships of negative correlations between ΔHbO and ΔHbR were observed in most channels. The Pearson’s product-moment correlation coefficients between ΔHbO and ΔHbR for left and right hand uses at each channel were calculated and denoted with green and blue numbers, respectively. All these values were statistically significant, where even the smallest value at Ch 17 for right hand use indicated a statistical significance of p < 10^−19^. This consecutive relationship between ΔHbO and ΔHbR over sessions indicate that a certain type of hemodynamics response reproducibly occurred in cortical regions through sessions at different dates ranged over several months.

## Discussion

For many years, signal contamination has been one of the major concerns for obtaining functional hemodynamic responses using the fNIRS technique in the human cerebral cortex^22,37^. A finding that application of pressure on the skin between an optode pair can eliminate signals measured with a conventional fNIRS^20^ clearly exhibited the existence of signal contamination from blood flow changes in the scalp layer. Removal of contamination from scalp blood flow is crucial for precise fNIRS measurements, including monkey studies. Several studies on the measurement of monkey neural activity using fNIRS have been reported^38–43^. In these studies, optodes were fixed at the dura^39,42,43^, at the scalp^38,41^, or were adhered to the skull^40^. These types of optode fixations were performed before each measurement, which can lead to infections and can impair positional reproducibility. As such, these methods are problematic for long-term continuous monitoring that is required for studies on neural plasticity in cerebral cortex. In this study, we developed a quickly detachable optode system with high inter-optode density on the skull surface thereby allowing highly reproducible and less-invasive multichannel fNIRS measurement over several months within a single subject.

In both session series, the time required for food retrieval increased when changing the food well size from its initial size. This suggests that picking food from a well of a new size was initially challenging regardless of whether the well size was increased or decreased. Following this initial adjustment phase, retrieval time decreased in all subsequent sessions (Fig. 7a). Presumably, the delayed retrieval time causes alterations in functional activation. Indeed, both maximum amplitude and the time of maximum amplitude of the hemodynamic response in the hand M1 area were positively correlated with the time required for food retrieval (Fig. 7b) and exhibited similar distributions in both hemispheres and both Hb species. This finding confirms that hemodynamic responses between hemispheres and Hb species obey predicable common relationships to neuronal activation.

We also observed that the hemodynamic responses in each motor-related region highly correlated with movement of the relevant body part. Activity was initially detected in the PMA, followed by that in the relevant hand M1 and mouth M1 areas. Several methods of determining causality such as dynamic causal modeling^44^ and Granger causality analysis^45^ have been applied to functional blood-oxygen-level-dependent MR images^46^ and fNIRS data^47,48^. Application of causality analysis using our fNIRS system may be quite useful in quantitating the relationships of motor cortex activity and movement proficiency.

After the series of experiments in this study, the subject monkey received an artificial cerebral infarct in a unilateral posterior limb of internal capsule using the method we had previously reported^49^. The subject showed a motor deficit in the contralateral hand of the lesioned hemisphere. After the recovery from the motor deficit through rehabilitative training of about two months, the fNIRS measurement during the food retrieval task was conducted. While the time for food retrieval by the contralateral hand of the lesioned hemisphere recovered to a level of no significant difference from those in the pre-lesioned condition, a part of PMA in the lesioned hemisphere newly increased hemodynamic responses during the retrieval and the statistical interaction between the factors of pre/post-lesion and left/right-hand-use showed significant difference in this region. On the other hand, in the pre-lesion condition even when data were obtained with an interval more than 2 months, such significant difference was not found in any channel (details will be reported elsewhere). We should note that cortical activity changes during recovery from brain damage could be evaluated by using our fNIRS system, which enables long-term reproducible monitoring of functional hemodynamic responses over motor related cerebral cortices.

Because the direct fixation of fNIRS optodes to the skull surface should not be allowed for human study, certain approaches will be required for eliminating artifacts caused by optodes fluctuation. Some commercially available fNIRS systems (NIRSport, NIRx Medical Technologies, USA, etc.) are equipped with improved optode devices of relatively more stable during subject’s motion. For more strict approach, some methods for motion artifact detection^17,18^ and for motion artifact removal^19^ are available. Using these improved devices and methods, fNIRS signals containing less motion artifacts will be measured even for human subject in free moving condition.

In order to remove the fNIRS signal contaminations from systemic physiological blood fluctuations in the scalp tissue, several methods have been proposed^21,50–54^. Especially, the hemodynamic modality separation (HMS)^21^ realized a real-time removal of contaminants other than the cerebral functional signal^55^. Based on many previous fNIRS studies, the HMS method theoretically assumed that the changes in oxygenated and deoxygenated Hb in the cerebral functional hemodynamics negatively correlate while those in other hemodynamics positively correlate. However, hemodynamics in the cortical tissue has never directly observed in human and non-human primates. In this study, ΔHbO and ΔHbR opposed one another (Figs 4 and 5), confirming the cerebral hemodynamic response reported by various fNIRS studies using skull-exposed rodents^56–59^ and human subjects under passive tasks or stimulations^60–65^. Moreover, the consecutive linear relationships between ΔHbO and ΔHbR were reproduced over several months at almost all channels (see Supplementary Fig. S2). These results confirm the theoretical principle of the HMS method. This finding indicates that contaminants in the conventional fNIRS signal mostly originate from the scalp tissue and that the HMS method effectively removes them even in human subjects. The use of the HMS method should make precise fNIRS measurements of functional hemodynamic responses in humans possible.

## Conclusions

In the present study, the hemodynamic responses in the motor-related cerebral region during food retrieval were successfully measured using an optimized fNIRS acquisition system. Because the ability to monitor brain activity was stable over days to months, it will enable future studies that monitor the neuroplastic changes that occur during brain damage recovery.

## Electronic supplementary material

Supplementary Information 
