# Supplementary material for: Functional near-infrared spectroscopy for monitoring macaque cerebral motor activity during voluntary movements without head fixation
Source: Sci Rep. 2018 Aug 9;8:11941. doi: 10.1038/s41598-018-30416-7 (PMC6085340; doi:10.1038/s41598-018-30416-7)
Supplement: Supplementary file 1 — Supplementary Information [file 41598_2018_30416_MOESM1_ESM.pdf]

Functional near-infrared spectroscopy for monitoring macaque cerebral motor activity during voluntary movements without head fixation.

Toru Yamada[1], Hiroshi Kawaguchi[1], Junpei Kato[1, 2], Keiji Matsuda[1], Noriyuki Higo[1]

[1]Human Informatics Research Institute, National Institute of Advanced Industrial Science and Technology (AIST).

[2]Graduate School of Comprehensive Human Sciences, University of Tsukuba.

Corresponding author:

Toru Yamada,

Human Informatics Research Institute, National Institute of Advanced Industrial Science and Technology (AIST), 1-1-1, Umezono, Tsukuba, Japan 305-8568

e-mail adress: toru.yamada@aist.go.jp.

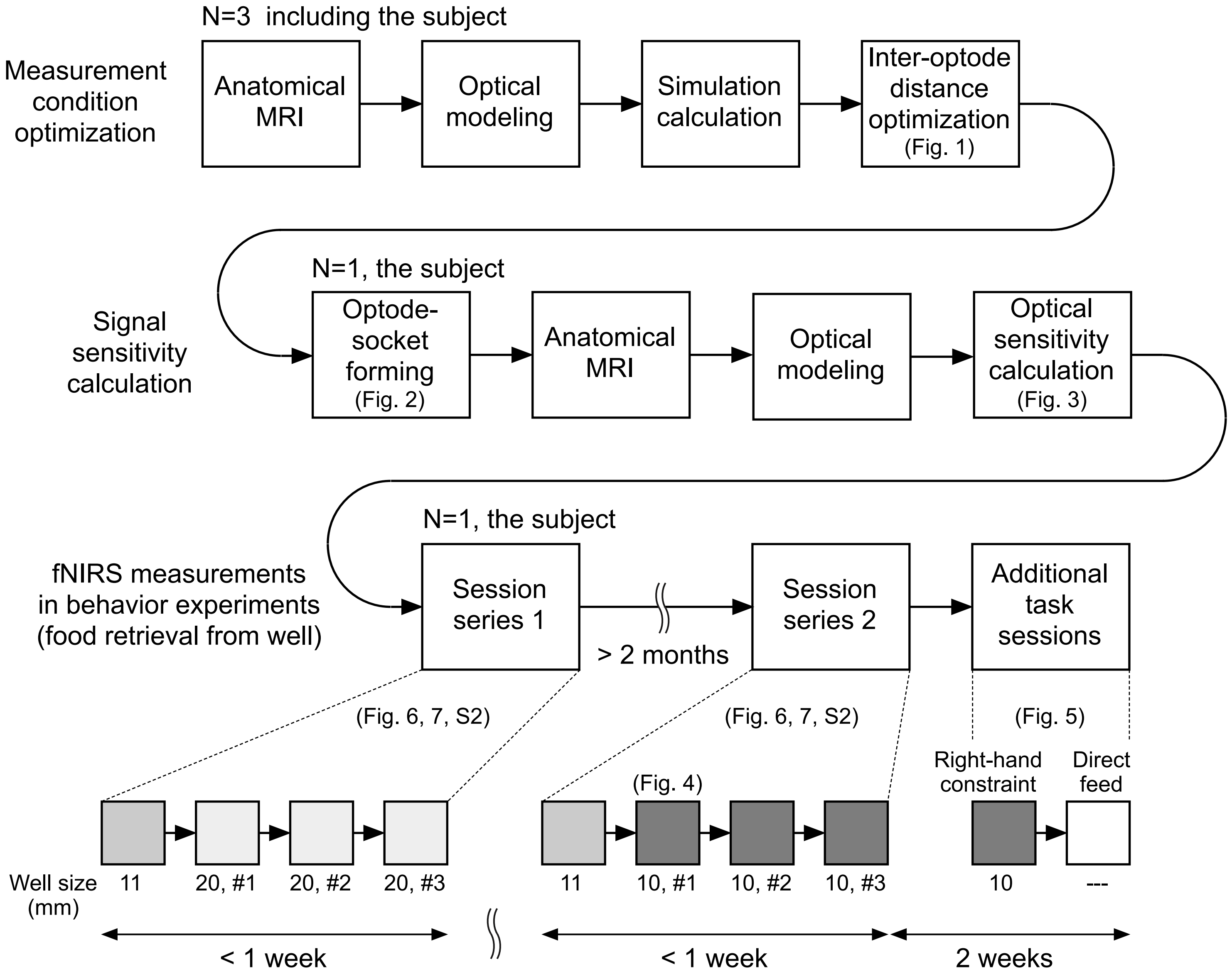

Fig. S1. Overview of this study. First, anatomical MRI data of three monkey subjects were used for optimization of the inter-optode distance. Second, for a single subject of the three subjects, optode-socket forming was conducted and fNIRS signal sensitivity was calculated. And third, for this subject, fNIRS measurements during behavior experiments were conducted. The experiments were comprised of two series of sessions and two additional task sessions. Experimental details were described in section 2.5.

Table S1: The correlation of the hemodynamic response in M1 with time required for food retrieval

	Maximum amplitude		Time to maximum amplitude	
	ΔHbO	ΔHbR	ΔHbO	ΔHbR
Left hemisphere	0.770*	0.715 †	0.844*	0.386
Right hemisphere	0.905**	0.640	0.668 †	0.404

†, $p<0.1$ (marginal); *, $p<0.05$; **, $p<0.01$

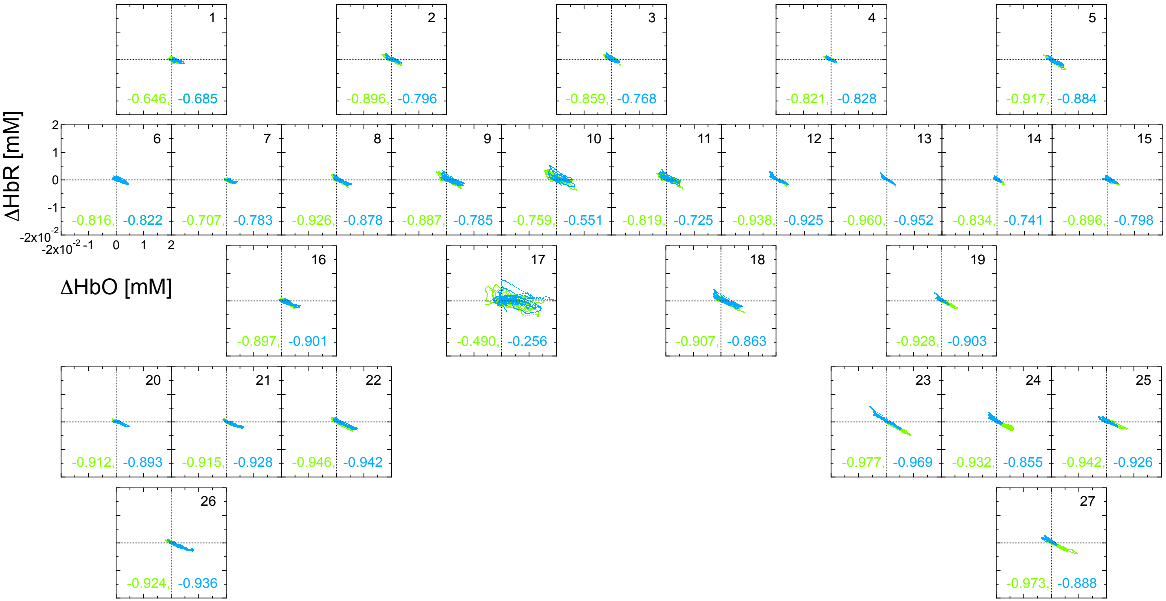

Fig. S2. Relationship between ΔHbO and ΔHbR during food retrieval. Each frame depicts the block averaged ΔHbO and ΔHbR of each channel among all sessions for left and right hand use plotted with green and blue dots, respectively.
